# Between Love and Exhaustion: A Qualitative Study of Greek Parents’ Lived Experiences Supporting Adult Children with Substance Use Disorders

**DOI:** 10.3390/nursrep15080306

**Published:** 2025-08-20

**Authors:** Panagiota Tragantzopoulou, Eleni Rizou

**Affiliations:** 1 School of Social Sciences, University of Westminster, 115 New Cavendish St., London W1W 6UW, UK; 2 Department of Applied Psychology, University of Derby, Kedleston Rd., Derby DE22 1GB, UK; e.rizou@mc-class.gr

**Keywords:** substance use disorder, parental caregiving, Greek families, qualitative research, stigma, collectivist culture, coping strategies

## Abstract

**Background/Objectives:** Parents of individuals with substance use disorders (SUDs) often carry significant emotional and relational burdens, yet their voices remain underrepresented in addiction research. This study explores how Greek parents navigate the long-term challenges of caring for adult children with SUDs, with a focus on emotional strain, caregiving identity, and culturally embedded coping strategies within a collectivist context. **Methods:** Eight Greek parents (six mothers and two fathers, aged 47–60) participated in in-depth, semi-structured interviews. Conversations were conducted either in person or via video call, depending on participant preference and geographical constraints. Data were analyzed using Interpretative Phenomenological Analysis (IPA) to explore lived experience and the meaning-making processes shaping parental coping over time. **Results**: Four overarching themes were identified as follows: (1) Living in Vigilance, reflecting constant hyper-alertness, emotional exhaustion, and social withdrawal rooted in trauma; (2) Shifting Parental Identity, capturing the evolution of parents into caregivers, advocates, and informal caseworkers amid systemic neglect; (3) Struggling Within Systems, highlighting exclusion, blame, and fragmentation in institutional care—with moments of empathy holding outsized emotional weight; and (4) Coping as Cultural Duty, showing how caregiving was sustained through values of sacrifice, loyalty, and protective silence, even at great personal cost. **Conclusions:** Greek parents supporting adult children with SUDs face a complex interplay of trauma, cultural obligation, and institutional strain. Their coping is shaped by deeply held familial values rather than access to effective support. The findings call for culturally attuned, family-inclusive interventions and further research into long-term caregiving across diverse contexts.

## 1. Introduction

Substance use disorders (SUDs) are a global public health concern with far-reaching emotional, relational, and social impacts that extend beyond the individual. Over 35 million adult family members worldwide are affected by the addiction of a close relative [1,2]. Despite this scope, addiction research has historically focused on the substance user, often neglecting close family members, particularly parents of adult children with drug problems [3,4]. This oversight is especially significant given the profound and enduring caregiving burdens many parents face, which diverge sharply from normative life-course expectations.

Parents of adult children with SUDs often experience a prolonged and emotionally complex caregiving role. Instead of supporting their child’s transition to autonomy, these parents often manage repeated crises, navigate fragmented services, and cope with chronic emotional strain [5]. The parent–child relationship is typically characterized by deep emotional ties, making disengagement more fraught compared to other familial relationships [6,7,8]. Moreover, societal expectations around parenting can amplify internalized guilt and shame, especially when the child’s condition is associated with socially stigmatized behaviors such as drug use [9,10]. Recent qualitative research further highlights the psychological toll of this caregiving role. Mothers of individuals in addiction recovery have reported experiencing ambiguous loss, profound emotional collapse, and long-term distress, often followed, though not inevitably, by a slow process of emotional recovery and posttraumatic growth [11]. 

In response to such challenges, the Stress–Strain–Coping–Support (SSCS) model has emerged as a robust framework to conceptualize the experiences of family members affected by a relative’s substance use. Developed in contrast to earlier models that pathologized the family, the SSCS model offers a more compassionate, relational understanding by focusing on how families respond to chronic stress [12,13]. The model highlights how drug-affected families experience significant psychological strain and must mobilize coping strategies and social support to manage the ongoing stress of their relative’s substance use. Coping strategies under the SSCS model are typically grouped into three broad categories: “standing up to” (efforts to regain control or change family dynamics), “putting up with it” (ranging from tolerance to passive resignation), and “withdrawing or gaining independence” (attempts to emotionally or physically distance from the user) [14]. These strategies reflect the difficult and often contradictory roles families must assume—balancing care and concern with self-preservation. Crucially, the model also draws attention to the importance of social support and how its presence or absence mediates strain.

The SSCS model has also underpinned the development of quantitative tools to assess family impact and coping, such as the Family Member Impact Scale and Coping Questionnaire, and has informed structured interventions to support affected family members [15,16,17]. These developments represent significant strides in legitimizing and addressing the experiences of those around the addicted individual, yet implementation remains uneven across services and cultural settings.

Despite growing recognition of the family’s role in supporting recovery, systemic and institutional barriers remain pervasive. Parents are frequently left navigating the addiction care system alone, with little professional guidance, inadequate support, and often no formal recognition of their caregiving role [18,19]. As a result, many delay or avoid seeking help until the situation becomes unmanageable, often relying initially on informal support networks that may themselves be limited by stigma or misinformation [20] The cumulative emotional, relational, and structural burdens placed on parents of adult children with SUDs highlight an urgent need for research that centers their perspectives. While some studies have indicated that perceived and social stigma can act as barriers to help-seeking among individuals with substance use issues [21,22], other research has found no significant association between stigma and help-seeking behavior [23,24]. These mixed findings highlight the complexity of help-seeking processes and suggest that stigma may operate in nuanced or indirect ways that warrant further investigation.

### Greece: Cultural Context and Family Coping

The cultural context in which substance use occurs plays a critical role in shaping how families respond to and cope with addiction. In collectivist societies, including Greece, emotional and physical detachment from a drug-using relative is often less acceptable. Seeking help outside the family unit may also be viewed as a failure of familial responsibility or loyalty [25,26,27]. Familism is especially pronounced in Mediterranean cultures, where individual identity is closely tied to the family collective, and where the preservation of family cohesion is paramount [28,29]. Key cultural indicators such as high levels of intergenerational cohabitation and strong parental involvement in adult children’s lives demonstrate the persistence of traditional familial norms, even within an increasingly modernized society [30,31]. The cultural concept of “one blood” [32] further underscores the collective sense of identity and responsibility that permeates Greek family life. This cultural backdrop has important implications for how coping strategies are developed and enacted by affected families.

According to the most recent nationwide survey conducted in 2004, 8.6% of individuals in Greece aged 12–64 reported having used an illicit substance in their lifetime, with cannabis being the most commonly used drug [33]. While this figure may appear relatively modest, it reflects a significant public health concern when extrapolated across the population. This concern is particularly heightened in a context where families serve as the primary source of care and intervention. Despite this evidence, relatively little research has focused on the lived experiences of families affected by drug use, particularly in terms of how they cope with the emotional, relational, and practical burdens of addiction. A study of Greek families coping with a child’s addiction highlighted the extensive emotional, financial, and relational strain experienced by affected family members [34]. Participants described a pervasive sense of disruption to family functioning, accompanied by feelings of guilt, isolation, and helplessness. Importantly, many parents remained actively involved in their child’s life—even in the face of long-term drug use and repeated relapses. This reflects deeply ingrained cultural values around familial duty and interdependence [34]. An additional qualitative study conducted in Greece revealed that both drug users and their families manage stigma through the culturally embedded practice of “not allowing the right”—a strategy aimed at controlling disclosure to protect family honor [35]. This underscores how stigma in Greece is not only individual but deeply familial, with parents often bearing a significant emotional burden. While the study offers valuable insight into stigma management within Greek culture, it included only a small number of parents and did not focus solely on their experiences. This limits its applicability in fully understanding parental coping, highlighting the need for further research centered specifically on parents.

As Karkanida [36] observes, Greek parents often reach out for external support only after becoming emotionally depleted, overwhelmed, and burdened by intense feelings of shame, anger, and failure. This emphasizes the limitations of relying solely on intra-familial coping strategies, especially in the absence of culturally attuned support services. Although observational studies and clinical reports indicate that families frequently act as key motivators for initiating treatment, their own needs and struggles remain underexplored. For instance, Pomini et al. [37], in their analysis of counseling sessions from a low-threshold agency in Athens, found that family members played a pivotal role in encouraging drug users to seek help. These findings are supported by other Greek practitioners [38,39], who emphasize the importance of involving families in treatment planning and delivery [40]. However, such findings stand in contrast to more recent qualitative research that highlights the significant barriers professionals face when working with families. A study by Misouridou & Papadatou [4] revealed that mental health professionals frequently experience their interactions with parents as emotionally taxing and conflictual, particularly in the early stages of practice. Challenges such as weak therapeutic alliances, poor communication, and inter-team tensions often led to disengagement or the referral of family members to other services. These difficulties raise important questions about systemic support for collaborative family work. Conversely, findings from a mixed-methods study by Passa & Giovazolias [41], which examined parents’ experiences in self-help groups within KETHEA’s therapeutic program, present a more hopeful narrative. Self-help, conceptualized as a collective, peer-based support process grounded in mutual aid and shared lived experience, was associated with reduced parental anxiety, improved coping, and healthier family communication. Notably, many parents shifted from patterns of silence and emotional withdrawal to more open dialogue after participating in the program. Taken together, these findings point to a gap between the recognized value of family engagement in treatment and the practical difficulties of implementing it within strained and under-resourced clinical contexts. Despite increasing recognition of parents’ dual roles as caregivers and affected individuals, significant gaps persist in our understanding of how they navigate the complex emotional and relational terrain of living with addiction.

In light of the limited body of qualitative research on family coping with addiction, the current study makes a valuable contribution by offering an in-depth exploration of how Greek parents manage the challenges of caring for a child with substance use problems. By centering parents’ lived experiences, the study shifts away from pathologizing or deficit-based perspectives and instead highlights the adaptive and culturally embedded strategies families use to endure and respond to addiction. This reframing is particularly important in the Greek context, where powerful cultural norms surrounding parental responsibility, family honor, and interdependence continue to shape how families experience and respond to drug use. In a society where the family functions as the central support structure, understanding how parents cope is critical—not only for informing service provision, but also for challenging dominant narratives and policies that overlook the family’s role and needs. This study was conducted for the purpose of exploring how Greek parents experience and make sense of caring for adult children with substance use disorders, with particular attention to emotional strain, caregiving identities, and culturally shaped coping strategies.

## 2. Materials and Methods

### 2.1. Design

This study employed a qualitative design informed by Interpretative Phenomenological Analysis (IPA), which is particularly suited to exploring how individuals make sense of complex, emotionally laden experiences [42]. Central to IPA is the double hermeneutic process, where participants interpret their lived experiences, and the researcher, in turn, interprets these interpretations [42]. This methodology allowed for an in-depth understanding of the emotional and relational dimensions of parental caregiving in the context of SUDs.

### 2.2. Participants

Following ethical approval, eight parents (six mothers and two fathers) of young adults living with an SUD were recruited for the study. Although the sample size (*n* = 8) is relatively small, it is consistent with established guidance for IPA [42], which emphasizes depth and richness of individual accounts over broad generalizability. Saturation was approached through iterative engagement with each transcript, where emerging themes began to repeat across cases, indicating a sufficient depth and breadth of experiential data [43]. The sample size was thus considered adequate for capturing both individual variation and shared patterns in parents’ caregiving experiences. Inclusion criteria included being a parent or legal guardian of a young person (aged 18–30) with a diagnosed or self-identified SUD, and having recent caregiving involvement (within the past 12 months). Exclusion criteria included being a professional caregiver or having a background in addiction-related healthcare, to preserve the focus on informal caregiving perspectives.

Participants were recruited through a purposive and snowball sampling strategy. The primary researcher distributed a digital flyer via social media channels, with interested individuals invited to contact the research team. Snowball sampling occurred through the parents themselves, who referred others in their networks and community-based peer support groups; however, none of the participants were known to the researchers prior to the study, and no pre-existing relationships were identified among participants. This approach helped preserve the independence and confidentiality of participants’ accounts. All participants were based in urban or semi-urban areas of Greece and identified as having an active, informal caregiving role in their adult child’s recovery journey. Their caregiving typically involved daily or near-daily emotional and practical support—such as crisis intervention, medication management, and advocacy—and had been sustained over periods ranging from 3 to over 15 years. Demographic information is presented in Table 1. To protect participant anonymity, all names used throughout the study, including those in the table below and direct quotes, are pseudonyms.

### 2.3. Data Collection

All participants provided informed written consent. Semi-structured interviews were conducted by the first author, a Greek psychotherapist with training in qualitative health research. Interviews took place either in person or online via Microsoft Teams, depending on participant preference and regional constraints. Data collection occurred between July and August 2024 and January and February 2025.

An interview guide was developed based on IPA principles and informed by the literature on family caregiving in mental health and substance use contexts. The guide was pilot-tested with two individuals familiar with caregiving roles (not study participants), and minor revisions were made for clarity and emotional sensitivity. The final guide included 10 open-ended core questions, each with optional follow-up prompts to encourage elaboration. Questions were designed to explore participants’ emotional experiences, family role shifts, interaction with systems of care, and personal coping strategies (see Table 2).

Interviews lasted between 60 and 100 min and were audio-recorded with consent. The tone was conversational and empathic, aligned with IPA’s emphasis on rapport and interpretative depth. Participants were encouraged to speak openly and emotionally, with reminders that there were no right or wrong answers. After each interview, field notes were recorded to capture contextual observations and reflexive insights.

### 2.4. Data Analysis

Interviews were transcribed verbatim by the first author and anonymized. The transcripts were then analyzed using IPA as outlined by Smith et al. [42]. Each transcript was read multiple times, with initial exploratory notes focusing on descriptive, linguistic, and conceptual features. Emergent themes were identified for each case individually before moving to cross-case analysis.

Themes were clustered into superordinate categories through iterative analysis. NVivo 12 software was used to assist with coding and theme organization. The development of themes was collaborative such that the first author generated initial interpretations, and the second author reviewed these for coherence and depth. Discrepancies were resolved through dialogue and consensus. Reflexivity was maintained throughout the research process, with both authors keeping reflexive journals to examine how their own experiences, assumptions, and social positions shaped the analytic process. Both researchers are Greek and have lived in Greece, with the second author residing there her entire life and the first having also spent several years living and working in the United Kingdom. Their shared cultural background provided insight into the Greek social and familial norms surrounding addiction, while also necessitating critical reflection to avoid over-familiarity or cultural assumptions. Professionally, both are female psychotherapists, with the first author having a background in qualitative research and the second possessing extensive clinical experience in addiction treatment. These perspectives informed the analysis and interpretation of the data, particularly in relation to the emotional, relational, and systemic dimensions of caregiving. Reflexive journaling supported awareness of these influences and helped mitigate potential biases. To further enhance trustworthiness, member checking was conducted with three participants, who reviewed the thematic summaries and confirmed that the themes accurately captured their experiences. This feedback did not lead to major modifications but reinforced the credibility and resonance of the interpretations.

### 2.5. Ethical Considerations

Ethical approval for the study was obtained from the Ethics Committee of the second author’s university (Protocol Code: 10056932; Approval Date: 10 July 2024). The study was conducted in accordance with the ethical principles outlined in the Declaration of Helsinki. Given the emotional sensitivity of the subject matter, all participants provided written informed consent prior to the interviews. Participants were reminded of their right to pause or withdraw from the study at any time without consequence. A resource sheet containing contact information for mental health and addiction support services was provided after each interview. No adverse incidents occurred during data collection. Ethical protocols concerning anonymity, confidentiality, and secure data storage were strictly followed throughout the research process.

## 3. Results

Four superordinate themes emerged from the Interpretative Phenomenological Analysis (IPA): (1) Living in Vigilance, (2) Shifting Parental Identity, (3) Struggling Within Systems, and (4) Coping as Cultural Duty. These themes capture the intense emotional landscape, evolving caregiving roles, cultural influences, and complex institutional interactions experienced by parents supporting a child with an SUD. Despite differences in background, all participants described a shared landscape of emotional labor, systemic fatigue, and unrelenting advocacy. A summary of how codes relate to each theme is presented in the Code–Theme Relationship Table below (Table 3) to enhance transparency. In the analysis that follows, numerical information (e.g., 6/8 participants) is included to indicate the prevalence of key experiences and enhance the clarity and evaluability of the findings. The narrative is further enriched by illustrative quotations from participants.

### 3.1. Living in Vigilance: The Emotional Weight of Constant Watchfulness

All participants (8/8) described living in a state of heightened emotional alertness—what several referred to as “living on edge” or “never switching off.” This was not a response to a specific incident but a chronic condition rooted in prolonged exposure to unpredictable crises:

*“You don’t sleep. You doze. Always listening—for a door, a creak, a phone call. Even silence makes you jump.”*

(Maria)

Seven participants (7/8) recounted a permanent state of emotional readiness: sleeping with phones on loud, avoiding travel, and restructuring their lives around the possibility of relapse, overdose, or disappearance. This vigilance led to anticipatory anxiety that blurred into trauma:

*“I once found him blue on the bathroom floor. Since then, I hear water running and my stomach drops.”*

(Anna)

Five parents (5/8) described becoming hyper-attuned to small behavioral cues. One mother likened it to *“developing a second nervous system”: “If he didn’t call at 5:05, I’d panic. My body knew before my brain did.”* (Elena). This constant watchfulness came at the cost of physical and psychological health. Words like “burned out,” “worn thin,” and “hollow” were common across seven accounts (7/8). The emotional exhaustion was compounded by invisibility—parents noted that while concern was directed toward their children, their own suffering was rarely acknowledged:

*“Everyone asks how he is. No one ever asks how I’m doing.”*

(Kostas)

Five participants (5/8) described withdrawing socially due to fatigue, shame, or the fear of being judged. One parent recalled avoiding family gatherings:

*“I stopped going to family dinners. Too many questions I couldn’t answer.”*

(Maria)

Living in Vigilance was emotionally paradoxical. Participants spoke of walking a tightrope—balancing love with fear, hope with dread. It was both a strategy for survival and a source of prolonged distress:

*“It’s like walking a tightrope in the dark. You don’t know when it’ll snap.”*

(Dimitra)

Summary: Living in Vigilance reflects the deep emotional toll of parenting in crisis mode. This theme highlights trauma-related hypervigilance, anticipatory anxiety, and social retreat—all rooted in a prolonged sense of uncertainty and fear.

### 3.2. Shifting Parental Identity: Between Parent, Nurse, and Advocate

All participants (8/8) described a transformation in their parenting identity. They no longer saw themselves solely as “mothers” or “fathers,” but as unpaid caseworkers, crisis responders, and health navigators. These new roles were not chosen—they were imposed by necessity:

*“I learned how to clean wounds, manage panic attacks, even administer meds. I’m a full-time carer, just without the title.”*

(Sophia)

Seven parents (7/8) expressed frustration at having to perform tasks they believed professionals should manage. They reported being forced to learn medical, psychological, and legal systems from scratch. Their previous lives were often upended:

*“I used to run a small business. Now I run around making appointments, fighting with clinics, and trying to keep my son alive.”*

(Petros)

Six participants (6/8) spoke of mourning the loss of a “normal” parent–child relationship. Their roles had become unidirectional, focused on containment rather than connection:

*“He used to call me ‘mum.’ Now it’s like I’m his caseworker. I miss being just his mum.”*

(Anna)

Ambivalence and guilt surrounded boundary setting. Four parents (4/8) enforced strict limits to protect their mental health, while others (4/8) admitted they struggled to say no—even at great personal cost:

*“You love them, but you have to protect yourself too. It took me years to understand that.”*

(Petros)

Many (5/8) described societal and professional scrutiny, saying they felt accused of either “doing too much” or “not enough.” This double bind intensified internal conflicts:

*“You’re either doing too much or too little. There’s no winning.”*

(Maria)

All participants (8/8) had developed extensive knowledge of healthcare and addiction systems—often out of sheer necessity. Some (4/8) even referred to themselves as “self-trained experts”:

*“Google became my best friend. I had to become fluent in addiction-speak to keep up.”*

(Dimitra)

Six parents (6/8) eventually became informal advocates, speaking at events, helping peers, or organizing support. Yet, this activism stemmed from exhaustion rather than empowerment:

*“I didn’t choose to be an activist. I just got tired of watching doors close in our faces.”*

(Anna)

Summary: Shifting Parental Identity captures the involuntary role expansion and emotional disorientation experienced by parents. Their caregiving evolved into a contested and emotionally taxing identity—shaped by necessity, grief, and marginalization.

### 3.3. Struggling Within Systems: Exclusion, Judgment, and Resistance

Parents’ accounts revealed a deep ambivalence toward health and social systems. While they relied on these institutions, most (6/8) felt alienated, ignored, or blamed by them. Only two participants (2/8) described care professionals as collaborative or compassionate:

*“The social worker just listened. That was enough. I didn’t feel invisible for once.”*

(Anna)

These positive interactions were rare—and described with reverence. More often, parents encountered rigid policies and emotional indifference. Five parents (5/8) said they were excluded from decision-making, especially under the guise of confidentiality:

*“They told me, ‘He’s an adult, we can’t tell you anything.’ But I was the one who found him unconscious.”*

(Sophia)

Poor inter-agency communication compounded the sense of chaos. Several participants described having to explain their child’s situation repeatedly to new professionals. One mother likened it to starting from scratch every time:

*“Every new doctor meant starting from scratch. There was no handover, no history.”*

(Elena)

Some (3/8) took on the task of documenting every step—maintaining spreadsheets, creating personal care timelines, and coordinating services alone:

*“I kept all his records, all his meds, dates, names. I knew more than his GP.”*

(Kostas)

Six parents (6/8) reported experiencing stigma or judgment in clinical settings. They spoke of condescending language, dismissive body language, or assumptions of blame:

*“A nurse looked at me like I was part of the problem. No one said it, but I felt blamed.”*

(Petros)

Despite these negative experiences, even minor gestures of recognition held deep emotional meaning:

*“One nurse called me after a tough night, just to say ‘we see you.’ That stayed with me.”*

(Dimitra)

Summary: Struggling Within Systems reveals the emotionally taxing paradox of seeking help from systems that simultaneously marginalize and blame. Parents’ resilience lay in learning to navigate these structures—even when excluded from formal roles in their child’s care.

### 3.4. Coping as Cultural Duty

Parents described coping with the ongoing stress and emotional turmoil of supporting a child with a substance use disorder as deeply rooted in cultural values of loyalty, sacrifice, and endurance. For many (5/8), coping meant continuing to provide emotional and material support despite the personal toll, framed not as passive acceptance but as an unwavering expression of love and responsibility:

*“He’s my child. What do you expect me to do—leave him in the street? I’ll keep helping until my last breath.”*

(Maria)

At times, parents attempted to set boundaries or step back temporarily for self-preservation or to encourage their child’s responsibility. These decisions were emotionally fraught and often accompanied by guilt and a sense of failing their duty:

*“I told him: no more money, no more lies. But then I cried for days. You feel like you’re killing your own child.”*

(Sophia)

*“We didn’t speak for six months. But I thought of him every day. I still kept track through his friends.”*

(Petros)

Despite these moments of withdrawal, fully detaching—whether emotionally or physically—felt almost unthinkable. Letting go was widely seen as morally wrong or unnatural, particularly within strong family bonds:

*“Even if I let go with my hands, I can’t let go with my heart. That’s not how we were raised.”*

(Anna)

Even amid exhaustion and grief, many parents expressed quiet pride in their persistence. Their endurance was more than survival; it was an integral part of their identity and cultural values:

*“You learn to live with heartbreak. That’s what being a Greek parent means sometimes.”*

(Maria)

This persistent support often coexisted with the heavy burden of stigma. Several parents chose to conceal the reality of their child’s struggles from others, protecting the family’s dignity and shielding their child from judgment. This silence was experienced not as denial but as a loyal and protective act:

*“No one knows except my sister. I’d rather carry the shame myself than have them look at him with pity.”*

(Elena)

Summary: Coping as Cultural Duty captures how Greek parents navigated the challenges of caregiving by embracing endurance, loyalty, and protective silence—strategies that may not always reduce stress but are deeply meaningful and reflect culturally shaped obligations.

## 4. Discussion

This study sought to explore how Greek parents cope with the long-term challenges of having an adult child with an SUD, shedding light on their lived experiences through an IPA lens. The findings illustrate the deeply relational and cultural dynamics that inform coping strategies, aligning in part with the Stress–Strain–Coping–Support (SSCS) model (Orford et al., 2010a) [14], but also pointing to specific cultural, emotional, and temporal nuances that warrant critical attention.

### 4.1. Coping Strategies: Cultural Inflection of SSCS Typologies

The three SSCS-derived coping typologies—“putting up with it,” “standing up to it,” and “withdrawing”—were evident in participants’ narratives, though their expression appeared shaped by familial norms and moral obligations rooted in cultural and relational contexts. “Putting up with it” often involved long-term emotional containment, continued financial support, and protective silence—actions some participants described not as passive endurance but as expressions of love, loyalty, and familial duty. These responses may reflect values associated with Mediterranean familism, such as collective responsibility and concern for family honor [28,32]. A comparable study in Brazil [44] found similar strategies, including secrecy, limiting the person’s exposure to others, and continued caregiving even in distressing circumstances—highlighting how shame and concern for reputation also shaped coping in that context. However, in contrast to the Greek participants, Brazilian families frequently cited religiosity and faith as central emotional resources, turning to God for strength and meaning. Despite Greece’s strong Orthodox Christian identity [45], none of the Greek parents in this study mentioned faith or spiritual coping, suggesting that while both cultures are collectivist, coping practices are not monolithic and may diverge in how cultural scripts are enacted.

The “standing up to it” strategy—typically characterized by attempts to assert boundaries—was usually adopted after extended periods of emotional depletion and frustration. Even then, it was often accompanied by ambivalence and guilt, suggesting a tension between the emotional need for self-protection and internalized expectations of parental steadfastness [9,10]. A study with female spouses of substance users in India [46] similarly found that “engaged” coping—such as making the husband take oaths, confronting the issue, and emotionally reacting to the substance use—was commonly employed. While this mirrors the active boundary setting found in the Greek sample, the Indian participants were spouses rather than parents, and their responses often included overt emotional expressions, accusations, and financial negotiations, suggesting that relational position may influence what coping strategies are developed.

“Withdrawal” was described most cautiously and rarely as a full disengagement. Participants who attempted emotional or physical distancing often remained psychologically invested, highlighting the complexity of detachment in the context of strong kinship bonds. In this sense, their experiences may resonate with the concept of ambiguous loss [11] and reflect the relational entanglements described in other collectivist settings [25]. While these coping strategies share features with the SSCS model, they were interpreted and enacted in ways that were closely tied to participants’ moral identities and culturally situated understandings of parental responsibility.

### 4.2. Chronic Vigilance and Shifting Parental Identity

The first theme, Living in Vigilance, reflects the sustained hypervigilance experienced by parents. Participants described a life structured around anticipatory anxiety, with trauma-like symptoms emerging from prolonged exposure to crisis. This confirms previous findings that families, particularly parents, often exist in a state of psychological readiness due to the unpredictability of addiction-related emergencies [11,14]. Our findings extend this by suggesting that hypervigilance is not merely a cognitive or emotional state but a deeply embodied experience, with one participant describing it as “developing a second nervous system”. Such expressions highlight the somatic toll of prolonged caregiving and unresolved threat. This chronic alertness often entailed a progressive role transformation. Many participants described how their parental role morphed into that of informal nurse, case manager, crisis negotiator, and advocate. An involuntary shift that echoes D’Aniello et al.’s [5] findings on the expanded caregiving burdens faced by parents of substance-using children. Yet, what stands out in this study is that this shift was experienced not just as an increase in responsibility, but as an erosion of parental identity. Participants mourned the loss of “just being a parent,” describing an identity consumed by fear, vigilance, and societal judgment. In this way, the relational toll of addiction was grieved not only as emotional fatigue, but as a form of symbolic or social death wherein meaningful connection was perceived as lost or fundamentally altered.

This is consistent with qualitative research on families affected by addiction, where relatives reported grieving their loved ones as “present but socially absent” and experiencing helplessness in the face of addiction’s trajectory [47]. Importantly, the data in the current study suggest that these role changes were not neutral but emotionally and morally charged. Parents spoke of guilt, ambivalence, and fear of public shame, all of which were intensified by cultural expectations surrounding parental responsibility. While Salonia et al. [46] identified codependent strategies such as denial, hyper-involvement, and emotional withdrawal in spouses of substance users, our findings suggest that these same strategies are entangled with deeper moral imperatives in parental relationships, particularly within collectivist cultures like Greece. Concepts such as “one blood” and intergenerational cohabitation [28,32] appear to constrain parents’ ability to emotionally detach or re-establish boundaries. As such, while the coping strategies may resemble those found in other relational contexts, the stakes may be different given that parenthood may be imbued with a life-long sense of moral duty and existential responsibility, which complicates attempts at self-preservation.

Moreover, the parents in this study often struggled to emotionally detach from their children, not merely due to affection, but because of deeply internalized cultural norms and the fear of moral or social judgment. This emotional entanglement was rarely met with adequate institutional support. Instead, parents found themselves navigating the ongoing crisis largely alone. Their grief over the deteriorating relationship with their child was intensified by healthcare systems that failed to recognize their suffering or offer targeted, family-focused interventions. The perceived invisibility of caregivers, despite their proximity to the addiction, contributed to a profound sense of abandonment and heightened psychological strain. These patterns mirror findings from Settley’s [48] review, which highlights that caregivers’ physical and emotional wellbeing is routinely neglected, even as the cumulative stress of caregiving leads to burnout, disrupted social ties, and mental health deterioration. Similarly, Maina et al. [47] emphasized the relational rupture and helplessness experienced by caregivers, compounded by a lack of addiction literacy and formal support.

Across studies, a consistent picture emerges. The burden of care is borne in isolation, with little recognition from professionals or systems designed to support recovery. This marginalization not only accelerates caregiver burnout but increases vulnerability to long-term psychological harm. The current findings thus reinforce the necessity for family-centered interventions that address both the psychological strain and structural invisibility experienced by caregivers. As Kaur et al. [49] and Asante & Lentoor [50] argue, structured caregiver guidance, advocacy support, and collaboration with healthcare providers can significantly alleviate caregiver distress. Yet, such supports remain underutilized. Without intentional efforts by health systems to understand and respond to the complex needs of family caregivers, the cycle of silent suffering is likely to persist.

### 4.3. Systemic Failures and the Double Bind of Help-Seeking

Our findings point to significant institutional barriers faced by parents, echoing concerns raised in the literature about the practical and emotional strain of engaging with formal services. Participants expressed deep ambivalence toward healthcare and addiction systems, which they simultaneously depended on and felt excluded by. This mirrors Karkanida’s [36] observation that Greek parents often reach out only after prolonged emotional depletion and internalized guilt, suggesting that help-seeking is often a measure of last resort rather than a proactive step. Despite their central role as caregivers, parents in this study described feeling invisible, blamed, or obstructed by confidentiality laws—barriers also identified in earlier clinical reports [37,39]. Their frustration was compounded by fragmented communication with professionals, weak therapeutic alliances, and limited involvement in treatment planning—findings that align with Misouridou & Papadatou’s [4] work on practitioner–family tensions in early-stage therapeutic encounters.

Notably, these difficulties may reflect not only structural shortcomings but also relational dynamics between mental health professionals (MHPs) and affected family members (AFMs). Although the importance of family involvement in addiction treatment is widely acknowledged in research and policy, clinicians may remain reluctant or ambivalent about engaging families. Recent work suggests that MHPs sometimes enter competitive or conflictual dynamics with relatives, particularly when familial narratives or needs appear to challenge clinical authority or idealized conceptions of the patient [51]. In such cases, therapeutic triangles between MHPs, individuals with addiction, and their families risk becoming sites of mutual misrecognition rather than collaboration. The accounts of parents in our study, many of whom described being kept at a distance or sidelined, illustrate the practical effects of such ruptures.

Yet, even minimal recognition, such as being listened to or consulted, was experienced as deeply validating by participants. These moments seemed to restore a sense of visibility and dignity, reinforcing the importance of relational attunement in family-inclusive care. Furthermore, while stigma and shame were significant barriers, many parents actively resisted these constraints. Like participants in Passa & Giovazolias’ [41] study of KETHEA self-help groups, some respondents in our study described moments of advocacy, disclosure, and boundary setting. This suggests that stigma is not a fixed outcome but a social force negotiated over time. Parents’ responses were shaped not only by cultural scripts of silence and honor, but also by their own emotional exhaustion and urgent need for connection. In this light, our findings both confirm and complicate dominant narratives of family withdrawal: rather than passive victims of stigma, many parents demonstrated active, if ambivalent, forms of resistance, adaptation, and meaning-making within an unsupportive institutional landscape.

### 4.4. Implications for Services and Theory

The findings confirm the relevance of the SSCS model while illuminating how Greek cultural scripts reshape its expression. The model’s core elements—strain, coping, and support—were all observed, but coping strategies were more emotionally entangled and less individualistic than typically described in Anglo-American contexts. Moreover, the absence of systemic family-oriented services meant parents had few avenues for support, reinforcing the urgent call for the introduction of culturally responsive, inclusive interventions [15,16]. 

This study thus substantiates and extends previous research by showing that familial adaptation to addiction is both psychologically complex and culturally situated. In Greece, where the family remains the primary site of caregiving and moral identity [11], coping cannot be understood solely through individual frameworks. Instead, it must account for collective norms, intergenerational obligations, and socio-institutional absences. In reflecting on these findings, it becomes clear that the parental experience of caregiving in addiction contexts is situated at the confluence of emotional trauma, role strain, and systemic marginalization. While previous research has emphasized either the psychological impact or the institutional dynamics of caregiving [11,46,47,51], this study bridges both dimensions, showing how chronic vigilance and identity strain are exacerbated, not alleviated, by current service structures.

To address the challenges identified in this study, several practice-oriented recommendations emerge that may help bridge the gap between caregiver needs and existing service provision in Greece. Practitioners have long emphasized the importance of involving families in treatment planning and delivery [38,39,40]. However, these calls are often undermined by persistent systemic and relational barriers. The current findings highlight how parents continue to feel excluded and undervalued within treatment systems, echoing previous studies that document weak therapeutic alliances, fragmented communication, and role confusion between professionals and families [4]. Although the importance of family involvement is widely recognized in both national and international policy frameworks [1,16,17], mental health professionals may remain ambivalent or resistant to such collaboration. To move beyond this impasse, addiction services must actively embed family-centered models of care across all levels of practice. Rather than treating families as secondary to treatment or as sources of conflict, they should be regarded as essential stakeholders with unique experiential insight.

The findings also highlight the need for improved professional training and supervision for mental health practitioners who work with families affected by addiction. Several parents in this study described feeling misunderstood, marginalized, or even blamed by professionals, echoing research showing that clinicians sometimes resist family involvement when it is perceived to challenge clinical authority or therapeutic direction [51]. To mitigate these relational ruptures, professionals should be offered regular supervision and training in family systems thinking, collaborative practice, and trauma-informed care. Facilitated reflection through models such as Balint groups may also help clinicians examine their own responses to caregiver involvement and avoid adversarial dynamics.

Equally important is the development of targeted support services that respond directly to the emotional and practical needs of caregivers. Participants in this research repeatedly articulated a sense of emotional exhaustion, chronic vigilance, and isolation—yet few had access to tailored interventions. Structured psychoeducational programs, stress-reduction workshops, and peer-led support groups could help address these gaps. The 5-Step Method, for example, has demonstrated effectiveness in supporting affected family members and could be adapted to better fit Greek cultural values and familial dynamics [52,53]. Without such interventions, caregivers are left to manage the cumulative strain of their roles alone, often at great psychological cost.

Finally, there is a need for culturally sensitive public awareness campaigns that address stigma and social shame associated with addiction and caregiving [54,55]. Parents in this study often delayed help-seeking due to fear of judgment or reputational harm, and many described internalizing cultural narratives that equated parental involvement with failure. Public campaigns must work to reframe addiction as a systemic and relational issue rather than a personal or familial moral failing. Normalizing caregiver involvement in the recovery process could reduce social isolation and empower families to seek help earlier and more confidently.

This study is not without limitations. The sample, while rich in emotional depth and experiential insight, was relatively small and demographically homogeneous, consisting of only eight participants—most of whom were mothers and all of whom were Greek. As caregiving is deeply shaped by cultural, gendered, and social scripts, this narrow demographic focus may limit the transferability of findings. Fathers, in particular, were underrepresented, which may have obscured gendered variations in caregiving roles and emotional expression. Moreover, the exclusively Greek sample limits the ability to explore how intersecting factors such as race, migration status, or socio-economic background may compound or mitigate caregiving strain.

Nonetheless, the study has several notable strengths. As a qualitative investigation grounded in IPA, it provides an in-depth, nuanced exploration of a population that remains largely invisible in both the Greek and international addiction literature. By centering the voices of Greek parents, the research surfaces culturally specific dynamics, such as familial obligation, moral duty, and intergenerational loyalty, that significantly influence coping and caregiving. These insights extend the applicability of existing models like the SSCS, while challenging service providers and policymakers to consider how addiction-related caregiving is experienced differently across cultural contexts. In doing so, the study contributes both empirical depth and theoretical nuance to our understanding of caregiving as a relational, moral, and structurally mediated experience.

As caregiving is shaped by cultural scripts, future research should explore how race, ethnicity, class, and migration status intersect with caregiving burden. Additionally, longitudinal research is needed to understand how caregiving identities shift over time, particularly in relation to treatment outcomes or relapse cycles.

## 5. Conclusions

Parental caregiving in the context of addiction should not be viewed as peripheral or informal. It is a central and enduring form of relational labor. Parents are not merely bystanders to their children’s journeys through addiction and recovery. Instead, they are co-survivors, often navigating this terrain without adequate institutional support or cultural permission to speak openly. The findings underscore the importance of framing caregiving not simply as a set of coping strategies but as a culturally and temporally situated identity project; one that is shaped by love, trauma, social silence, and enduring resilience. Future research should extend these findings by exploring how factors such as gender, class, migration status, and religion further shape caregiving experience. Further, future research could adopt longitudinal designs to examine how parental coping and caregiving identities evolve over time, particularly in relation to treatment trajectories, relapse cycles, or the aging of both parents and their adult children. Comparative cross-cultural studies could also help distinguish which coping patterns are culturally specific and which are more universally experienced. Moreover, exploring the voices of fathers, siblings, and extended family members would further expand our understanding of how addiction reverberates through family systems in different cultural contexts. Only through such critical, contextualized understandings can services truly support the caregivers who remain at the heart of familial and societal responses to addiction.

## Figures and Tables

**Table 1:** Participant demographic characteristics.

Participant	Gender	Age	Relationship to Child	Child’s Age	Type of Substance	Region	Education Level	Employment Status
**Maria**	Female	53	Mother	24	Heroin	Athens	Secondary education	Part-time cleaner
**Elena**	Female	49	Mother	19	Cannabis/Cocaine	Patra	University degree	School administrator
**Kostas**	Male	56	Father	21	Heroin	Athens	Vocational diploma	Unemployed
**Dimitra**	Female	60	Mother	26	Alcohol/Heroin	Thessaloniki	Primary education	Retired
**Anna**	Female	52	Mother	23	Cocaine	Athens	University degree	Self-employed
**Sophia**	Female	47	Mother	20	Cannabis	Athens	Secondary education	Receptionist
**Petros**	Male	55	Father	25	Cocaine	Volos	University degree	Civil servant
**Christina**	Female	51	Mother	22	Methamphetamine	Athens	Secondary education	Homemaker

**Table 2:** Interview questions.

	Interview Questions	Sample Follow-Up Prompts
**1**	Can you tell me about when you first realized your child was struggling with substance use?	What were your initial thoughts or feelings? How did others in the family react?
**2**	How would you describe your role in your child’s life now?	Has that role changed over time? In what ways?
**3**	What has been the most emotionally challenging part of supporting your child?	Can you give an example? How did you cope with it?
**4**	What does a typical day look like for you as a caregiver?	Are there routines or tasks you always do?
**5**	How has this experience affected your own wellbeing—emotionally, socially, or physically?	Have you received support for yourself?
**6**	What has your experience been like with health or social care services?	Have there been moments that stood out—either helpful or frustrating?
**7**	Have your relationships with friends or family changed during this time?	In what ways? Why do you think that is?
**8**	How has your family’s background or values influenced the way you’ve approached your child’s struggles with substance use?	Have any expectations or beliefs shaped your decisions or emotions during this time?
**9**	How do you manage or cope on difficult days?	Are there specific strategies you use?
**10**	How do you see yourself and your role as a parent today, compared to when this journey began?	Have your priorities, identity, or relationships changed in ways you didn’t expect?

**Table 3:** Code–Theme Relationship Table.

Theme	Representative Codes	Participant Frequency
**1. Living in Vigilance**	- Constant emotional alertness	8/8
	- Fear of relapse or overdose	7/8
	- Disrupted sleep patterns	5/8
	- Trauma memories	5/8
	- Hyperawareness of behavioral cues	7/8
	- Social withdrawal	5/8
	- Tightrope metaphors for emotional tension	7/8
**2. Shifting Parental Identity**	- Parent as nurse, caseworker, and advocate roles	8/8
	- Emotional ambivalence (love vs. resentment)	4/8
	- Grieving loss of “normal” parent–child relationship	6/8
	- Learning to navigate medical, psychological, and legal systems	8/8
	- Lack of professional support	7/8
	- Struggles with boundary setting	4/8
	- Experiencing societal/professional scrutiny	6/8
**3. Struggling Within Systems**	- Systemic fragmentation and poor inter-agency communication	6/8
	- Exclusion from decision-making due to confidentiality	5/8
	- Recounting traumatic histories repeatedly	5/8
	- Feeling judged or blamed by professionals	6/8
	- Moments of rare validation and empathy	6/8
	- Parents as informal case managers/documenters	3/8
**4. Coping as Cultural Duty**	- Persistent emotional and material support despite personal cost	5/8
	- Protective silence and concealment to avoid stigma	5/8
	- Guilt and ambivalence when setting boundaries	4/8
	- Temporary withdrawal paired with ongoing emotional connection	4/8
	- Moral and cultural resistance to full detachment	3/8
	- Endurance as a source of identity and pride	5/8

## Data Availability

The data for this study can be made available upon reasonable request to the correspondent author (P.T.).

## Public Involvement Statement

No public involvement in any aspect of this research.

## Guidelines and Standards Statement

This manuscript was drafted using the COREQ (Consolidated Criteria for Reporting Qualitative Research) for qualitative research.

## Abbreviations

The following abbreviations are used in this manuscript:

SUDs	Substance use disorders
SSCS	Stress–Strain–Coping–Support
IPA	Interpretative Phenomenological Analysis
